# Clinical spectrum and factors impacting outcome of *Candida auris:* a single center study from Pakistan

**DOI:** 10.1186/s12879-019-3999-y

**Published:** 2019-05-06

**Authors:** Muneeba Ahsan Sayeed, Joveria Farooqi, Kausar Jabeen, Safia Awan, Syed Faisal Mahmood

**Affiliations:** Section of Infectious Diseases, Department of Medicine, Aga Khan University, Stadium Rd, Karachi, 74800 Pakistan

**Keywords:** Candida auris, Candidemia, urinary tract infection, Empyema, Colonization, Mortality

## Abstract

**Background:**

An outbreak of *Candida auris* began globally in 2014 including Pakistan and since then it has emerged as a nosocomial multi-drug resistant pathogen. The aim of this study was to assess the clinical spectrum and outcome of patients, from a single center in Pakistan, in whom *C. auris* was isolated.

**Methods:**

A retrospective study was conducted on 92 patients; ≥16 years with at least one culture positive for *C. auris,* at the Aga Khan University Hospital Karachi, Pakistan from Sept 2014-Mar 2017.Demographics, clinical history, management and outcome were studied. A logistic regression model was used to identify the risk factors for mortality.

**Results:**

We identified 92 patients with *C. auris* (193 isolates), of whom 52.2% were males. Mean age was 54.14 ± 20.4 years. Positive cultures were obtained after a median hospital stay of 14 days. Most patients had a history of surgery (57.6%), antibiotic use (95.6%), ICU stay (44.6%), indwelling lines (88.04%) and isolation of another multi-resistant organism (52.2%).Most patients were symptomatic (70.7%). Amongst these, 38 had candidemia while 27 had non-candidemia infections. Sites of infection included central lines (35), urinary tract (19), peritonitis (4), nosocomial ventriculitis (1), empyema (1), fungal keratitis (1) otitis externa (1) and surgical site (1). Fluconazole resistance was 100% while 28.5 and 7.9% were Voriconazole and Amphotericin resistant respectively. Overall crude mortality was 42.4% while 14-day mortality was 31.5%. Both infected and colonized cases shared similar mortality (46.2% vs 33.3%; *p*-value = 0.25). Among infected cases mortality was high in candidemia compared to non-candidemia (60.5% vs 25.9%) in which deaths related to *C. auris* were 34.2% vs 22.2% respectively. On multivariate analysis candidemia (AOR 4.2, 95% CI: 1.09–16.49; p-value = 0.037) was associated with greater mortality with source control being the only protective factor for mortality (AOR 0.22, 95% CI: 0.05–0.92; p-value0.038] while ICU stay, rapidity of blood culture clearance, DM, malignancy and MDR co-infection had no impact.

**Conclusion:**

Patients with *C.auris* from a single center in Pakistan have a wide clinical spectrum with line associated infection being the predominant site of infection. Candidemia leads to high mortality while source control improves outcome.

## Background

*Candida auris* has emerged as an antifungal resistant yeast causing invasive infections in nosocomial settings. Improved diagnostics and epidemiological typing techniques are essential to identify and characterize these strains [1, 2]. It was first reported in Japan in 2009 when it was isolated from external ear canal [3]. Later in 2011 it was reported for the first time as human pathogen causing nosocomial fungemia in South Korea and extremes of age with prior history of surgery was identified as a risk factor [4].*C. auris* was found to be closely related to *Candida haemulonii* and was reported to be misidentified as *C. haemulonii* using Vitek 2 YST and Phoenix (BD). There are numerous other misidentifications using the commercially available diagnostic methods. API 20CAUX misidentifies it as *Rhodotorula glutinis* and *C. sake.* Vitek 2 (bioMe’rieux) misidentifies it as *C. haemulonii, C. lusitaniae* and *C. famata* while MicroScan (Beckman Coulter Pasadena, CA) misidentifies this as *C. famata*, *C. lusitaniae*, *C. guilliermondii*, *C. parapsilosis*, *C. albicans* and *C. tropicalis* [2]. This wrong identification delayed its diagnosis, a finding later endorsed by *Kathuria S* et al in 2015 [5]. It is notable for its antifungal resistance creating a treatment dilemma [6–8].

In September 2014, Aga Khan University Hospital, Karachi, Pakistan experienced an outbreak of a yeast which was initially identified as *Saccharomyces cerevisiae* [9]*.* Because of the unusual antifungal susceptibility pattern of this isolated yeast, these isolates were retested at the Centers for Disease Control and Prevention (CDC), Atlanta, USA for identification and were eventually identified as *C. auris.* Simultaneously this pathogen was reported from India, South Africa and Venezuela and came to lime light [9]. So far, the available literature has emphasized on its methods of identification, antifungal susceptibility and risk factors associated with acquisition. Studies have shown that factors like extremes of age, presence of co-morbidities and prior antifungal therapy are associated with its acquisition while limited data is available on its disease spectrum and outcomes. Therefore, in this study, we have reported its clinical spectrum, outcomes and outcome determining factors among those with invasive infection. The aim of this study was to assess the clinical spectrum and outcome of patients, from a single center in Pakistan, in whom *C.auris* was isolated.

## Methods

We had conducted a retrospective study of 92 hospitalized patients at the Aga Khan University Hospital (AKUH), a 600-bed tertiary care referral hospital located in Karachi, Pakistan. All patients with *C. auris* positive culture from September 2014–March 2017 were enrolled. Patients aged < 16 years or those who did not receive primary treatment at AKUH were excluded.

Cultures were processed by standard operating procedures in use by the microbiology laboratory as part of initial clinical work up. Any yeast from a sterile sample was identified on the basis of germ tube test, appearance on chromogenic medium BiGGY (Oxoid), cycloheximide tolerance, microscopic morphology on Cornmeal Agar (Dalmau method), and profile number on API 20C AUX (bioMe’rieux). If identification was inconclusive (less than 90% confidence on API), the antifungal susceptibility results for Fluconazole and the API number generated were taken into account. *C. auris* was presumptively identified by a combination of certain features: resistance to fluconazole, absence of pseudo hyphae on thin Cornmeal Tween80 agar, obtaining profile numbers 2,000,130, 2,000,173, 2,102,173, 6,102,173 on API 20C AUX (bioMe’rieux, France). This phenotype had been verified by D1-D2 sequencing of 28S subunit of rDNA performed on the first 15 isolates from our institute sent to the Mycotic Diseases Branch, CDC, Atlanta. There was 100% concordance between the described phenotype and sequencing results. Antifungal susceptibility was tested using SensititreYeastOne (Trek Diagnostic Systems Ltd., East Grinstead, England) and E-test (bioMe’rieux SA, Marcy l’Etoile, France). Susceptibilities against fluconazole, voriconazole, and amphotericin B were available for all isolates. For an additional 28 isolates MIC values were also available against itraconazole, posaconazole, anidulafungin, micafungin and caspofungin. The susceptibilities were interpreted for *Candida* species according to CLSI M27-S3 for triazoles and echinocandins and M27-A3 guidelines for amphotericin B [10, 11]. Briefly, based on these, the isolate was considered resistant to fluconazole if MIC was ≥64 μg/ml, voriconazole if MIC was ≥4 μg/ml, non-susceptible to amphotericin if MIC was > 1 μg/ml, echinocandins if MIC was > 2 μg/ml. isolates were considered susceptible if MIC to fluconazole was ≤8 μg/ml, voriconazole was ≤1 μg/ml, amphotericin was ≤1 μg/ml, and echinocandins was ≤2 μg/ml.

Demographic data including age, gender, date of admission, unit type and a brief history including risk factors (such as diabetes mellitus, malignancy, prior history of surgery, prior antibiotics or antifungal exposure, prior ICU stay, indwelling lines and history of isolation of any other multi-resistant organism), site of infection and outcomes were collected using data collection forms. Data was collected by the primary author only. Patients with *C. auris* in cultures and associated clinical sign and symptoms (including but not limited to fever, site specific symptoms and relevant laboratory parameters such as white blood cells and C-reactive protein) were labeled as symptomatic/infected. On the other hand, patients lacking associated clinical signs and symptoms with *C. auris* isolated from non-sterile sites were considered asymptomatic/colonized. In case of multiple positive cultures from the same patient the date of first positive culture was considered as the date of infection/colonization. The site of infection was labeled using CDC definitions [12]. As candida is excluded from CDC’s definition for urinary tract infections (UTI), this was defined as clinical sign and symptoms of a UTI with positive urine culture in the presence of urinary WBC and absence of epithelial cells. Asymptomatic candiduria was defined as presence of *C. auris* in urine without clinical sign and symptoms. Standard criteria were used for the definition of diabetes mellitus and malignancy. Use of prior antimicrobials was defined as administration of any antibiotic/ antifungal in last 90 days of positive blood culture. Isolation of other multi-drug resistant (MDR) bacteria before isolating *C. auris* was defined as bacteria that were resistant to more than 3 classes of antibiotics, isolated in last 90 days of positive blood culture for *C. auris* while co infection with MDR bacteria was their isolation within 7 days of positive culture for *C. auris*. Whenever the given antifungal agent was in standard-weight based dosage according to site of infection, and when the isolate was reported as susceptible to it on culture, antifungal therapy was considered appropriate. This is in accordance with our institute’s antifungal policy. For all invasive candida cases the first line empiric antifungal therapy was intravenous Amphotericin B deoxycholate 0.75 mg/kg. Lipid formulations of amphotericin B were not used. Voriconazole was considered in cases with deranged renal functions when amphotericin could not be administered. It was given in a dose of 6 mg/kg 12 hourly on day 1 followed by 4 mg/kg 12 hourly. Therapeutic drug monitoring for voriconazole was not available. Early appropriate antifungal therapy was the appropriate antifungal therapy given in ≤48 h while late appropriate antifungal therapy was started 48 h after positive culture. Source control was defined as the removal of inciting agent or focus and entailed the removal of central line in case of Central Line Associated Blood Stream Infection (CLABSI), Foley’s catheter in Catheter Associated Urinary Tract Infection (CAUTI), exploratory laparotomy in peritonitis, drainage of pus collection and removal of lumbar drain in ventriculitis. Early source control was the removal of inciting agent in ≤48 h while late source control was when it was achieved after 48 h of positive culture. Only crude mortality rate (CMR) was assessed. Microbiological failure was considered in candidemia patients and was defined as failure to clear blood culture within 4 days while clinical failure was defined as 14-day mortality or microbiological failure. We considered 14-day mortality as there were large number of patients who lost to follow-up during the retrospective chart review. In patients who did not survive, cause of death was also assessed by physicians on clinical and laboratory evidence, and was thought to be related to *C. auris* when other causes of death were excluded and patient had an active *C. auris* infection at that time.

We sought ethical approval from University’s Ethics Review Committee (ERC number 4381-MED-ERC-2016). The committee waived the requirement for informed consent. To preserve confidentiality, we coded each patient and removed their original identifications.

The collected data were analyzed through Statistical Package for the Social Sciences (SPSS), version 22.0. For continuous data, mean and standard deviation/ median with interquartile range were reported depending on normality assumption. For categorical data frequency and percentage were reported. In order to assess association of mortality with independent variables Chi square test was applied and *P* value < 0.05 was considered significant. Variables for univariate analysis were based on known risk factors of mortality for invasive candidiasis and on the preliminary analysis of our ongoing outbreak study on C. *auris* [9, 13]. All statistically significant and clinically relevant variables of univariate analysis were included in a multivariate logistic regression analysis to determine the independent predictive factors of mortality among infective *C. auris* cases. Results were given as odds ratios (OR) and 95% confidence intervals (95% CI).

## Results

**Fig. 1:**
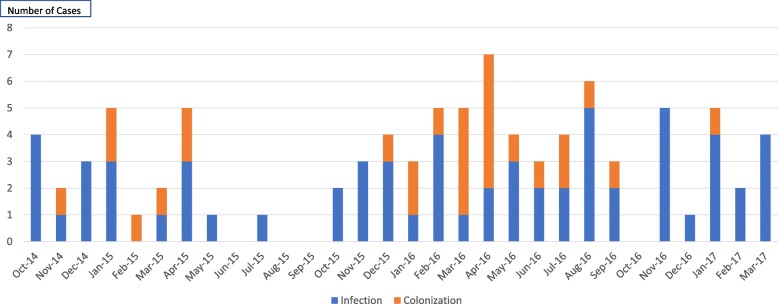
EPI-Curve of Candida *Auris* cases

From 2014 to 2017, 193 *C. auris* strains were isolated from 92 patients with 16.3% confirmed as *C. auris* by D1-D2 sequencing while rest were presumed to be *C. auris*. The number of cases with *C. auris* rose over the study period from 13 patients in the last quarter 2014 to 46 in 2016 (Fig. 1: Epi-curve of *C. auris* cases). Table 1 describes the baseline characteristics of the patients. The median days from admission to positive culture was 14 (IQR-7-130). Most of the patients were older (median age 55.5 years), were currently or previously admitted to an Intensive Care Unit/ High Dependency Unit (ICU/HDU), had indwelling lines (central lines and drains), and received antibiotics. One in three patients (39.1%) had also received antifungals in last 90 days of positive culture. Fifty-two percent had MDR bacteria isolated from different sites prior to *C. auris* while 31.5% had a concomitant MDR bacterial infection.

**Table 1:** Demographics of 92 patients with *Candida auris*

Category	Total *n* = 92 (100%)
Male	48 (52.2%)
Age group	
16–35 years	20 (21.7%)
36–55 years	26 (28.3%)
56–75 years	30 (32.6%)
76–96 years	16 (17.4%)
Location in hospital	
ICU	30 (32.6%)
Special care	30 (32.6%)
Ward	27 (29.3%)
Outpatient	5 (5.4%)
Diabetes mellitus	26 (28.3%)
Malignancy	17 (18.5%)
Surgery	53 (57.6%)
Abdominal	12 (13%)
Cardiothoracic	8 (8.7%)
Neurosurgery	14 (15.2%)
ENT	9 (9.8%)
Orthopedics	7 (7.6%)
Others	2 (2.17%)
ICU stay in last 30 days	41 (44.6%)
Last ICU stay duration (weeks) ≤ 2 Weeks	31 (75.6%)
HDU stay in last 30 days	62 (67.4%)
Last HDU stay duration (weeks)	
≤ 2 Weeks	52 (56.5%)
Indwelling lines	81 (88.04%)
Antibiotics in last 90 days	88 (95.6%)
Antifungals in last 90 days	36 (39.1%)
Fluconazole	19 (20.7%)
Voriconazole	3 (3.3%)
Amphotericin	7 (7.6%)
> 1 Antifungal	7 (7.6%)
Prior antifungal duration (weeks)	
≤ 2 Weeks	25 (27.2%)
Isolation of multi-drug resistant bacteria	
Prior to*C.auris* (≤ 90 days)	48 (52.2%)
Coinfection with *C.auris*	29 (31.5%)
Infected cases	65 (70.7%)
Candidemia	38 (58.5%)
Non-candidemia	27 (41.5%)
UTI	19
Peritonitis	3
Nosocomial ventriculitis	1
Empyema	1
Surgical Site Infection	1
Otitis externa	1
Keratitis	1
Colonized cases	27 (29.3%)
Asymptomatic candiduria	21
Central line tip	4
Ear Swab	1
Oral Swab	1
Antifungal resistance (*n* = 63)	
Fluconazole	63 (100%)
Voriconazole	18 (28.57%)
Amphotericin	5 (7.93%)
Mean hospital stay (days)	30.73
Median hospital stay (days)	25 (1–163)
Mortality	39 (42.4%)
*C auris* death	19 (48.7%)
14-day mortality	29 (31.5%)
Mean days in which mortality occurred (days)	12.41
Median days in which mortality occurred (days)	7 (1–69)
Clinical failure	38 (41.3%)
Microbiological failure (Candidemia)	11 (40.74%)

Antifungal susceptibility was checked in isolates from 63 patients (68.4%) in which fluconazole resistance was seen in all, voriconazole resistance in 18 (28.6%) and amphotericin resistance seen in 5 (7.9%) patients. Isolates from 3 patients (4.76%) were resistant to 2 classes of antifungals (azoles and polyenes) while none were resistant to all classes tested. No echinocandin resistance was detected.

Of the 193 *C. auris* strains, majority were isolated from blood (75/193) and urine (83/193). Out of 83 urine specimens 73 were from indwelling catheters, while 10 were mid-stream urine samples. Only 21 patients (22.8%) isolated *C. auris* from 2 or more sites.

Of the 92 patients, 65 (70.7%) were infected while 27 (29.3%) were colonized with *C. auris*. Amongst the infected patients, the most common site of infection was the bloodstream accounting for 38 (41.3%) cases, most of which were CLABSIs (35, 38.4%). Of the CLABSI patients, 9 (25.71%) were complicated by seeding to different organs causing endovascular thrombosis or empyema. Source of the fungemia could not be identified in 2 patients. Among non-candidemia patients, UTI was the most commonly seen site infected (19 of 27 patients) with 11 having CAUTI and 8 non-catheter associated UTI.

Of the 65 infected patients, 55 were treated with antifungals, amongst which only 44 patients (67.7%) received the appropriate antifungal therapy. Early appropriate antifungal therapy was administered to 20 (45.5%) patients. Amongst the patients who received appropriate antifungal therapy, majority received amphotericin alone (42, 95.5%) while 2 patients were treated with voriconazole alone. However, 7 patients who initially received amphotericin were later switched to voriconazole. Simultaneous dual antifungal therapy was not given to any patient.

Out of 38 patients with candidemia, 31 (81.57%) received appropriate antifungal therapy for a mean duration of 18.8 days. Only amphotericin was used to treat candidemia. Early appropriate antifungals were administered in 16 (51.6%) candidemia patients. However, mortality was higher in those receiving early antifungals (11 of 16, 68.7%) compared to those who received antifungals late (7 of 15; 46.7%). There were 7 patients who did not receive antifungal therapy, of which 2 cleared their candidemia with source control alone. Culture clearance was achieved in 25 out of 27 patients in whom clearance was checked. However, microbiological failure (i.e. lack of clearance within 4 days) was seen in 11 patients. Clearance could not be checked in 10 of the remaining 11 patients due to early mortality.

Amongst the 27 non-candidemia patients, 22 received antifungal, of which 13 were appropriate. In particular, of the 19 patients with UTI, 10 received appropriate antifungal therapy for a mean duration of 12 days. In 8 (72.7%) of the 11 patients with CAUTI, the catheter was removed. Five patients did not receive any antifungal, out of which 2 died within 24 h of the positive culture. Patients with keratitis received topical and systemic amphotericin while otitis externa was managed with clotrimazole ear drops. Compared to non-candidemia, candidemia patients had a higher mortality rate (60.5% vs. 25.9%; *p*-value = 0.006) and higher 14-day mortality (44.7% vs. 22.2%; p-value = 0.019).

Urine was the most common site of *C. auris* isolation in asymptomatic patients (21/27) of whom 17 were catheterized. While the catheter was removed in 10 of these patients, repeat cultures were sent in 2 patients only, both of which were negative. Table 2 compares characteristics and outcomes of infected and colonized *C. auris* cases. There was no difference in gender, age distribution, location in hospital, co-morbidities, co-infection with MDR bacteria and probability of dying between the two.

**Table 2:** Comparison of *C. auris* infected patients with *C. auris* colonizers

Variable	Infection (*n* = 65)	Colonization (*n* = 27)	p-value
Male	35 (53.8%)	13 (48.1%)	0.85
Age group			
16–35	15 (23.1%)	5 (18.5%)	
36–55	20 (30.8%)	6 (22.2%)	0.51
56–75	21 (32.3%)	9 (33.3%)	
76–95	9 (13.8%)	7 (25.9%)	
ICU	23 (35.4%)	7 (25.9%)	0.12
HDU	24 (36.9%)	6 (22.2%)	
Ward	11 (40.7%)	16 (24.6%)	
DM	18 (27.7%)	8 (29.6%)	0.85
Malignancy	13 (20%)	5 (18.5%)	0.80
Surgery	39 (60%)	14 (51.9%)	0.61
Co-infection with MDR bacteria	22 (33.8%)	7 (25.9%)	0.45
Appropriate antifungal therapy	44 (67.7%)	0	< 0.001
Source control	40 (61.5%)	7 (25.9%)	< 0.001
Mortality	30 (46.2%)	9 (33.3%)	0.25
14-day mortality	23 (35.4%)	6 (22.2%)	0.45
Clinical failure	24 (64.9%)	6 (22.2%)	< 0.001
Alive	35 (53.8%)	18 (66.7%)	
*C. auris* death	19 (29.2%)	0	
*Non-auris* death	11 (16.9%)	9 (33.3%)	0.005

The median length of hospital stay in all patients was 25 days (IQR 1–163). The CMR was 39 (42.4%) in which 19 deaths (48.7%) were related to *C. auris*. The 14-day mortality was 29 (31.5%) and the median days in which mortality occurred was 7 (IQR 1–69).

Table 3 shows details *C. auris* infective cases and mortality impacting factors*.* The mean days in which mortality occurred was 5.26 days and 14- day mortality rate was 94.7%.Compared to the infected survivors, these patients were more likely to be older (56–75 years, AOR 4.5, 95%CI:1.1–18.9) and had candidemia (AOR 4.2, 95%CI: 1.1–16.5). Survivors were also more likely to have had adequate source control (AOR 0.2, 95%CI: 0.1–0.9).

**Table 3:** univariate & multivariate analysis to determine the risk factors for mortality among infective *C. auris* cases

Category	Died *n* = 30 (100%)	Survived *n* = 35 (100%)	Univariate		Multivariable	
			OR*[95% CI]	*P*-value	AOR^†^[95% CI]	*P*-value
Age group						
16–35	5 (16.7%)	10 (28.6%)	1.0			
36–55	10 (33.3%)	10 (28.6%)	2.0 [0.5–7.9]	0.32		
56–75	13 (43.3%)	08 (22.9%)	3.2 [0.8–13]	0.09	4.5 [1.09–18.9]	0.038
76–96	02 (6.7%)	07 (20%)	0.5 [0.08–3.8]	0.56		
Location in hospital						
ICU	14 (46.7%)	09 (25.7%)	3.4 [0.8–13]	0.07	1.2 [0.24–5.8]	0.81
Special Care	11 (36.7%)	13 (37.1%)	1.86 [0.5–7.0]	0.35		
Ward	05 (16.7%)	11 (31.4%)	1.0	0.19		
Outpatient	0	02 (3.1%)				
Diabetes Mellitus	11 (36.7%)	07 (20%)	2.3 [0.7–7.0]	0.13		
Malignancy	06 (20%)	06 (17.6%)	0.96 [0.20–3.2]	0.95		
Candidemia	23 (76.7%)	15 (42.9%)	4.3 [1.4–12]	0.007	4.2 [1.09–16.49]	0.03
Early blood clearance (≤72 h)	02 (18.2%)	5 (35.7%)	1.0			
Late blood clearance (> 72 h)	09 (81.8%)	9 (64.3%)	2.5 [0.38–16.42]	0.34		
No blood clearance	02 (15.4%)	0 (0)				
Appropriate antifungal therapy	23 (76.7%)	21 (60%)	2.2 [0.7–6.4]	0.15		
Early antifungal therapy	14 (46.7%)	6 (17.1%)	4.66 [1.2–17.43]	0.02	0.84 [0.16–4.3]	0.83
Late antifungal therapy	09 (30%)	15 (42.9%)	1.2 [0.3–4.09]	0.77	4.1 [0.94–17.9]	0.06
Source control	17 (42.5%)	25 (57.5%)	0.52 [0.18–1.46]	0.2	0.22 [0.05–0.92]	0.038
Early source control	07 (41.2%)	13 (56.5%)	1.0			
Late source control	10 (58.8%)	10 (43.5%)	1.5 [0.4–5.3]	0.49		
Co infection with multidrug resistant bacteria	13 (43.3%)	09 (25.7%)	2.2 [0.77–6.2]	0.13	2.7 [0.61–12.6]	0.18

*OR, odds ratio

†AOR, adjusted odds ratio

## Discussion

We describe the clinical characteristics and outcomes of patients in whom *C. auris* was isolated in a single center in Pakistan. We found a wide clinical spectrum ranging from asymptomatic patients to invasive disease including candidemia, urinary tract infections, empyema and ventriculitis. Most of the infections occurred in critically ill patients who were admitted for a prolonged period of time.

The crude in-hospital mortality was 42.4% which is similar to the mortality rate seen in other studies ranging from 35.2–60% [9, 14]. A total of 31.5% of the deaths were related to *C. auris* infection, majority of whom died within the first week of acquiring this. This high mortality rate is in contrast with the UK outbreak reported by Schelenz S*etal* where no deaths were directly attributable to *C. auris* [15]. As expected candidemia patients had a higher mortality, though interestingly, antifungal therapy did not make a difference in the mortality. Also, paradoxically we found that mortality was higher in those who received early antifungal treatment; however this is likely reflective of the severity of their illness which prompted rapid initiation of amphotericin which is in contrast to other studies [6, 16]. Moreover, we could not check if this difference was statistically significant due to the small numbers involved. Mortality was also higher in the older patients, which has been shown in several other studies [15, 17, 18]. The most important factor impacting outcome was adequate source control. This is in line with established treatment guidelines for treating invasive candidiasis [19]. On the other hand, contrary to other studies, diabetes and malignancy were not associated with mortality in our study, possibly due to small numbers [14].

We found an annual increase in the number of identified cases at our institute. While patients with *C.auris* are managed with barrier precautions, single room isolation is not possible due to space constraints. Similarly, costs preclude obtaining environmental and patient surveillance cultures. As a result, clonal spread of our strains of *C.auris* has been documented and highlights the challenges of infection prevention in resource limited countries [9].

Despite skepticism regarding the propensity of *C. auris* to cause urinary tract infection or empyema as stated by CDC, we have found that UTI was the second most common site for symptomatic infection [20]. Moreover, we describe several unusual infection sites including wound, peritoneum, otitis externa, keratitis, ventriculitis and empyema. However, as expected bloodstream infection, in particular CLABSI was the most common infection seen.

The strength of our study is the large number of patients with comprehensive clinical data, giving a better understanding of the spectrum of disease caused by *C.auris*. Moreover, this is the largest series looking at the gamut of infections caused by the South Asian strain of *C.auris* in both ICU and non-ICU patients. However, our study has several limitations. Firstly, the data was retrospectively collected leading to potential information bias. Secondly, and perhaps more importantly, not all of our *C. auris* isolates were confirmed by sequencing. However, the characteristic phenotype we have described had 100% concordance in the 15 strains in which D1-D2 sequencing was performed. Similarly, we were unable to perform genetic antifungal resistance testing. However, of the 15 strains from Pakistan which underwent whole genome sequencing 2 distinct ERG 11 hotspot mutations were identified: Y123F and K143R [9].

## Conclusion

In conclusion, *C.auris* is associated with a wide variety of invasive infections and carries a high mortality rate. Source control is the most effective therapeutic intervention to reduce mortality in these critically ill patients.

## Abbreviations

95% CI: 95% Confidence Interval
AAF: Appropriate antifungal
AKUH: Aga Khan University Hospital
AOR: Adjusted odds ratio
C. *auris*: Candida auris
CAUTI: Catheter Associated Urinary Tract Infection
CDC: Centers for Disease Control and Prevention
CLABSI: Central Line Associated Blood Stream Infection
CMR: Crude mortality rate
DM: Diabetes mellitus
HDU: High dependency unit
ICU: Intensive care unit
IQR: Interquartile range
MDR: Multi drug resistant
MR: Mortality rate
OR: Odds ratio
SPSS: Statistical Package for the Social Sciences
UTI: Urinary Tract Infection
